# Lack of adipocyte IP3R1 reduces diet-induced obesity and greatly improves whole-body glucose homeostasis

**DOI:** 10.1038/s41420-023-01389-y

**Published:** 2023-03-09

**Authors:** Xin Zhang, Lu Wang, Yubo Wang, Linjuan He, Doudou Xu, Enfa Yan, Jianxin Guo, Chenghong Ma, Pengguang Zhang, Jingdong Yin

**Affiliations:** 1 State Key Laboratory of Animal Nutrition, College of Animal Science and Technology, China Agricultural University, 100193 Beijing, China; 2 Molecular Design Breeding Frontier Science Center of the Ministry of Education, 100193 Beijing, China

**Keywords:** Metabolic disorders, Type 2 diabetes, Homeostasis

## Abstract

The normal function of skeletal muscle and adipose tissue ensures whole-body glucose homeostasis. Ca^2+^ release channel inositol 1,4,5-trisphosphate receptor 1 (IP3R1) plays a vital role in regulating diet-induced obesity and disorders, but its functions in peripheral tissue regulating glucose homeostasis remain unexplored. In this study, mice with *Ip3r1* specific knockout in skeletal muscle or adipocytes were used for investigating the mediatory role of IP3R1 on whole-body glucose homeostasis under normal or high-fat diet. We reported that IP3R1 expression levels were increased in the white adipose tissue and skeletal muscle of diet-induced obese mice. *Ip3r1* knockout in skeletal muscle improved glucose tolerance and insulin sensitivity of mice on a normal chow diet, but worsened insulin resistance in diet-induced obese mice. These changes were associated with the reduced muscle weight and compromised Akt signaling activation. Importantly, *Ip3r1* deletion in adipocytes protected mice from diet-induced obesity and glucose intolerance, mainly due to the enhanced lipolysis and AMPK signaling pathway in the visceral fat. In conclusion, our study demonstrates that IP3R1 in skeletal muscle and adipocytes exerts divergent effects on systemic glucose homeostasis, and characterizes adipocyte IP3R1 as a promising target for treating obesity and type 2 diabetes.

## Introduction

The increasing prevalence of obesity and type 2 diabetes worldwide has led to increased complications of cardiovascular diseases, hypertension, fatty liver disease, and cancer [1, 2]. With the global spread of severe acute respiratory syndrome coronavirus 2 (SARS-CoV-2), obesity and related metabolic disorders accelerate severe COVID-19 [3]. Obesity-induced ‘unhealthy’ white adipose tissue (WAT) displays a pro-inflammatory state and enhanced fibrosis and hypoxia, leading to the onset and progression of type 2 diabetes [4], while numerous studies also showed no association between adipose inflammation and metabolic dysfunction or insulin action [5, 6]. Furthermore, skeletal muscle contributes to approximately one-third of postprandial glucose disposal [7, 8], and its diminished response to insulin is characteristic of type 2 diabetes. Therefore, studies are urgent to clarify factors that modulate WAT and skeletal muscle functions in controlling glucose homeostasis, especially in the development of obesity.

Calcium is a critical second messenger regulating gene expression, protein synthesis, muscle contraction, and metabolism [9]. Accumulating evidence has identified disturbing calcium signaling as emerging factor involved in the insulin resistance development [10, 11]. IP3Rs are ubiquitous ligand-gated Ca^2+^ release channels located on the membrane of endoplasmic reticulum (ER). Their important roles have been recognized for neurological, immunological, cardiovascular, and neoplastic human diseases by identifying specific mutations [12]. Strikingly, IP3R1 heterozygous mutant mice were susceptible to diet-induced glucose intolerance and insulin resistance [13]. The reduction of IP3R1-mediated ER-mitochondria Ca^2+^ transfer conduces to the development of complications of type 2 diabetes, including diabetic cardiomyopathy and hepatic insulin resistance [14, 15].

Growing evidence has highlighted the association between IP3R1 expression level and functions of skeletal muscle and adipose tissue. For example, IP3R1 expression was reduced in the skeletal muscle of aged mice, and its inhibition was detrimental to muscle regeneration [16]. Regarding WAT, up-regulation of CD36 in preadipocytes was reported to induce lipid accumulation and inflammation through activating IP3R1 [17]. Of particular importance is that muscle dysfunction and inflammation account for the progression of insulin resistance [18, 19]. Thereby, it can be anticipated that the contribution of IP3R1 to glucose homeostasis may be different in skeletal muscle and WAT. However, it remains obscure whether and how IP3R1 affects whole-body glucose homeostasis by balancing the functions in different tissues, especially in WAT and skeletal muscle.

In this study, we observed the abnormal regulation of IP3R1 in the diet-associated insulin resistance. We generated two mouse models lacking IP3R1 selectively in skeletal muscle or adipocytes to decipher its role in maintaining whole-body glucose homeostasis and identified adipocyte IP3R1 as a potential therapeutic target to promote metabolic health during over-nutrition.

## Results

### Identification of IP3R1 as a regulator of diet-induced insulin resistance

**Fig. 1:**
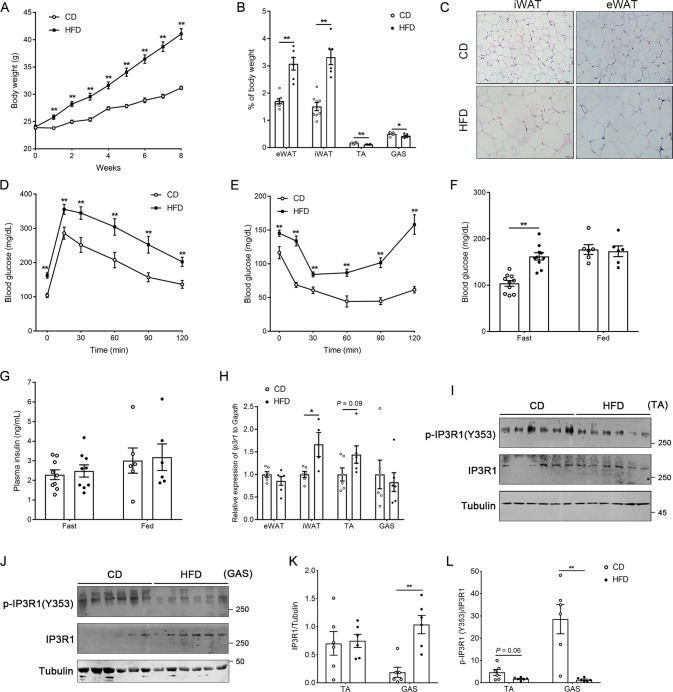
Metabolic analysis and IP3R1 expression of C57BL/6 mice on chow diet (CD) or high-fat diet (HFD). **A** Body weight of mice maintained on CD or HFD for 8 wk (*n* = 15). **B** Tissue weight (eWAT, iWAT, TA and GAS) percentage of body weight (*n* = 6-7). **C** Representative HE-stained sections of iWAT and eWAT from mice fed CD or HFD (*n* = 6-7). Scale bar = 50 μm. **D** GTT (2 g/kg glucose, i.p.) (*n* = 10). **E** ITT (1 U/kg insulin, i.p.) (*n* = 10). **F**, **G** Blood glucose and plasma insulin levels under fasting and fed states (*n* = 9-10 for fasting state, *n* = 6 for fed state). **H** Relative mRNA expression levels of *Ip3r1* in adipose tissues (iWAT and eWAT) and skeletal muscles (TA and GAS) (*n* = 6). **I**–**L** Western blot analysis of p-IP3R1 (Y353) and IP3R1 in TA and GAS lysates. Quantification of p-IP3R1 (Y353)/IP3R1 and IP3R1 was determined by ImageJ software (*n* = 6). Data were shown as means ± SEM. ^*^P < 0.05, ^**^P < 0.01. **A**–**C**, **F**–**L**: two-tailed unpaired Student’s *t* test; **D** and **E**: two-way ANOVA followed by Bonferroni’s post hoc test.

To investigate the potential role of IP3R1 in diet-induced obesity and metabolic disorders, C57BL/6 mice received a regular chow diet (CD) or a high-fat diet (HFD) for 8 wk. HFD dramatically increased the body weight of mice, elevated the mass of epididymal WAT (eWAT, visceral fat) and inguinal WAT (iWAT, subcutaneous fat), declined the mass of tibialis anterior (TA) and gastrocnemius (GAS), and also induced the expansion of adipocyte size (Fig. 1A–C). Furthermore, HFD led to metabolic disorders, including compromised glucose tolerance (Fig. 1D) and insulin sensitivity (Fig. 1E), and increased blood glucose levels under fasting condition (Fig. 1F). Notably, the HFD significantly increased *Ip3r1* expression in iWAT (*P* < 0.05), and tended to increase *Ip3r1* expression in TA (*P* = 0.09), while did not alter *Ip3r1* expression in eWAT and GAS compared with the control (Fig. 1H). We also found a significant increase in IP3R1 protein level in GAS (*P* < 0.01), but its phosphorylation on Tyr353 was significantly decreased in GAS (*P* < 0.01) and tended to decrease in TA (*P* = 0.06) in HFD group (Fig. 1I–L). Therefore, HFD increased IP3R1 expression but decreased its activation by tyrosine phosphorylation especially in the skeletal muscle.

### Generation of skeletal muscle-specific *Ip3r1* knockout mice

**Fig. 2:**
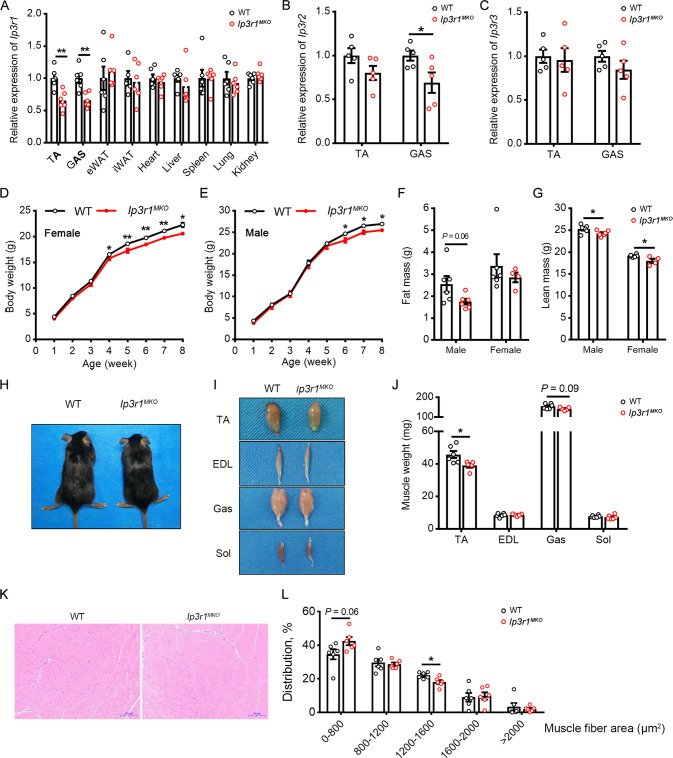
*Ip3r1* deletion in skeletal muscle leads to lower muscle weight. **A** mRNA expression levels of *Ip3r1* in skeletal muscle (TA and GAS), adipose tissues (eWAT and iWAT), heart, liver, spleen, lung and kidney (*n* = 6). **B**, **C** Relative mRNA expression levels of *Ip3r2* and *Ip3r3* in TA and GAS isolated from WT and *Ip3r1*^*MKO*^ mice (*n* = 5). **D**, **E** Body weight curve of WT and *Ip3r1*^*MKO*^ female and male mice (*n* = 10). **F** Fat mass and (**G**) lean mass of adult WT and *Ip3r1*^*MKO*^ female and male mice measured by the nuclear magnetic resonance system (*n* = 4–6). **H**, **I** Representative images of size and TA, EDL, GAS and Sol muscles in adult WT and *Ip3r1*^*MKO*^ mice. **J** Muscle weight of adult WT and *Ip3r1*^*MKO*^ mice (*n* = 5). **K** H&E staining of TA muscles and (**L**) frequency histogram of fiber cross-sectional area (*n* = 6). Scale bar = 100 µm. All data were analyzed by two-tailed unpaired Student’s *t* test and presented as means ± SEM. ^*^P < 0.05, ^**^P < 0.01.

Since skeletal muscle accounts for ~30% of postprandial glucose disposal, we aimed to investigate whether *Ip3r1* knockout in skeletal muscle affects glucose homeostasis. qPCR results revealed a significantly diminished *Ip3r1* level in TA and GAS of *Ip3r1*^*MKO*^ mice (*P* < 0.01), while *Ip3r1* expression was unaltered in other tissues (Fig. 2A). Further, *Ip3r1* knockout reduced *Ip3r2* expression in the GAS but not TA (*P* < 0.05, Fig. 2B). No changes were observed in *Ip3r3* expression levels in TA and GAS between genotypes (Fig. 2C).

*Ip3r1*^*MKO*^ mice grew slowly during postnatal growth, resulting in lighter body weights from week 4 or week 6 for female or male mice, respectively (Fig. 2D, E). MRI scanning demonstrated that *Ip3r1* conditional loss in skeletal muscle reduced lean mass of male and female mice significantly (*P* < 0.05), and tended to reduce the fat mass of male mice (*P* = 0.06) (Fig. 2F, G). Congruously, *Ip3r1*^*MKO*^ mice exhibited a smaller body size and decreased weight of TA (*P* < 0.05) and GAS (*P* = 0.09) at 8-week-old (Fig. 2H–J). Interestingly, we found that *Ip3r1* loss in skeletal muscle tended to increase the proportion of small muscle fiber (0–800 µm^2^, *P* = 0.06), while significantly decreased the proportion of large muscle fiber (1200–1600 µm^2^, *P* < 0.05, Fig. 2K, L). The shift of muscle fiber size distribution also evidenced that *Ip3r1* loss in skeletal muscle led to decreased muscle mass.

### Metabolic analysis of *Ip3r1*^*MKO*^ mice

**Fig. 3:**
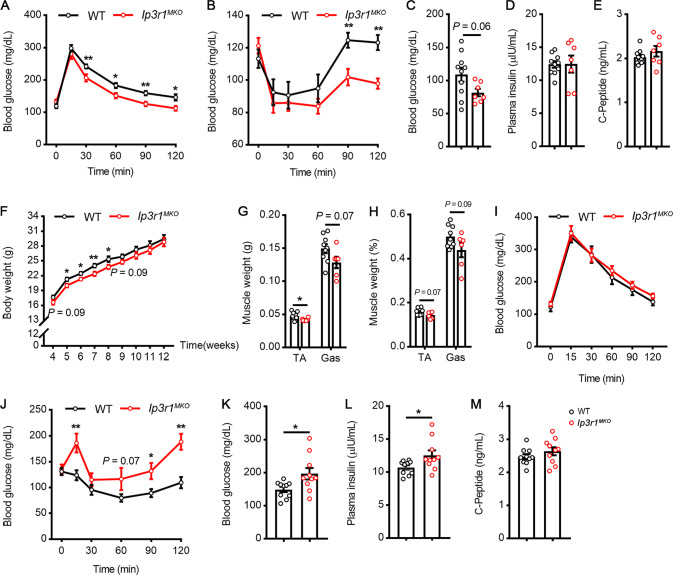
Role of IP3R1 in skeletal muscle in controlling systemic glucose homeostasis of mice maintained on chow diet (CD) or high-fat diet (HFD). **A** GTT (2 g/kg glucose, i.p.), **B** ITT (1U/kg insulin, i.p.), **C** blood glucose levels, **D** plasma insulin levels, and **E** plasma C-peptide levels after 8 wk of CD feeding (*n* = 7–10). **F** Growth curve of body weight during the 8 wk of HFD feeding (*n* = 10-11). **G** Muscle weight, **H** muscle weight percentage, **I** GTT (2 g/kg glucose, i.p.), **J** ITT (1U/kg insulin, i.p.), **K** blood glucose levels, **L** plasma insulin levels, and **M** plasma C-peptide levels after 8 wk of HFD feeding. *N* = 6–9 for **G** and **H**, *n* = 10-11 for **I** and **J**, and *n* = 10 for **K**–**M**. All Data are presented as means ± SEM. ^*^P < 0.05, ^**^P < 0.01. **A**, **B**, **I**, **J**: two-way ANOVA followed by Bonferroni’s post hoc test; **C**–**H**, **K**–**M**: two-tailed unpaired Student’s *t* test.

To access the metabolic roles of IP3R1 in skeletal muscle, WT and *Ip3r1*^*MKO*^ mice were first maintained on a regular chow diet. Results revealed that *Ip3r1*^*MKO*^ mice exhibited improved glucose tolerance and insulin sensitivity at 8-week-old, concomitant with the decreased blood glucose level in the fasting state (*P* = 0.06), although plasma insulin and C-peptide levels were similar between the two groups (Fig. 3A–E).

WT and *Ip3r1*^*MKO*^ mice at 4 wk of age were then challenged with HFD for 8 wk. *Ip3r1*^*MKO*^ mice recorded fewer body weights than WT mice (Fig. 3F), partly due to the lower muscle weight (Fig. 3G, H). Next, to determine the effect of skeletal muscle-specific *Ip3r1* deletion on HFD-induced metabolic deficits, a series of metabolic tests were performed. *Ip3r1* deficiency in the skeletal muscle caused a worsening of HFD-induced insulin resistance (Fig. 3J), along with a significant elevation in blood glucose levels and plasma insulin levels in the fasting state (Fig. 3K, L). Meanwhile, the two groups of mice showed similar glucose tolerance (Fig. 3I) and C-peptide level in plasma (Fig. 3M). Further, the expression levels of genes related to skeletal muscle fiber-type (*Myh7*, *Myh2* and *Myh4*) and mitochondrial function (*Pgc1α*, *Ndufs1*, *Ndufv2*, and *Cycs*) were not changed in TA and GAS (Supplementary Fig. S1A, B), while *Atp5a1* level was significantly increased in the TA of *Ip3r1*^*MKO*^ mice (*P* < 0.05, Supplementary Fig. S1A). AMPK signaling, the hub of metabolic control, was also detected. *Ip3r1* specific knockout in skeletal muscle significantly increased the phosphorylation of AMPK in TA (*P* < 0.01, Supplementary Fig. S1C, D) but not GAS muscle (Supplementary Fig. S1E, F), while WT and *Ip3r1*^*MKO*^ mice showed similar phosphorylation levels of ACC in TA and GAS muscle (Supplementary Fig. S1C–F). These data suggest different roles of skeletal muscle IP3R1 in maintaining glucose homeostasis under normal energy and energy-excess conditions.

### IP3R1 regulates the IR-Akt-GSK3β axis in skeletal muscle

**Fig. 4:**
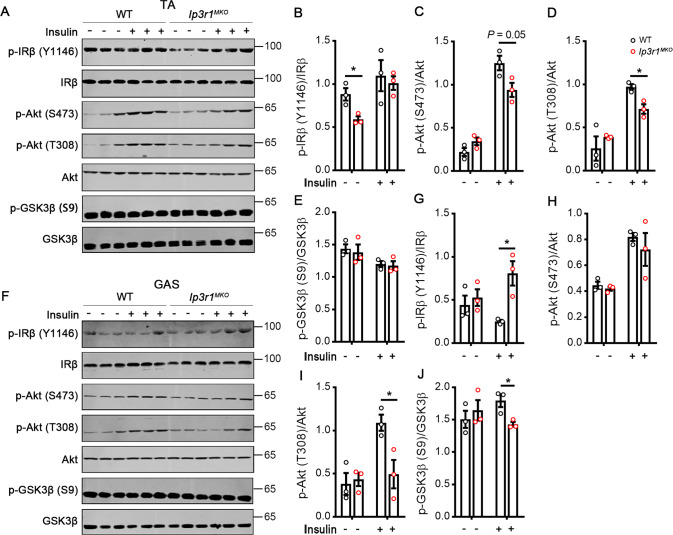
Mice with *Ip3r1* knockout in skeletal muscle show significantly reduced IR-Akt-GSK3β axis when maintained on a high-fat diet. Western blot analysis of p-IRβ (Y1146), p-Akt (S473), p-Akt (T308), p-GSK3β (S9), IRβ, Akt, and GSK3β in response to insulin (1 U/kg for 10 min) in (**A**–**E**) TA and (**F**–**J**) GAS lysates. Quantification of p-IRβ (Y1146)/IRβ, p-Akt (S473)/Akt, p-Akt (T308) /Akt, and p-GSK3β (S9)/GSK3β was determined by ImageJ software (*n* = 3). All data were analyzed by two-tailed unpaired Student’s *t* test and presented as means ± SEM. ^*^P < 0.05.

Insulin exerts a fundamental role in glucose homeostasis through Akt signaling pathway. Hence, we hypothesized that *Ip3r1* deficiency in skeletal muscle might affect insulin-stimulated Akt activation when mice were exposed to excessive energy intake. To this end, WT and *Ip3r1*^*MKO*^ mice were fed an HFD for 8 wk and intraperitoneally injected with insulin (1 U/kg body weight). After 10 min, TA and GAS from mice under basal and insulin-stimulated states were sampled. Under the basal state, except for the phosphorylation of IRβ tyrosine residue, the phosphorylation of IRβ, Akt, and Akt target GSK3β in TA and GAS was not changed between the groups (Fig. 4). As expected, the insulin-stimulated p-Akt (S473) (*P* = 0.05) and p-Akt (T308) (*P* < 0.05) levels were blunted in the TA of *Ip3r1*^*MKO*^ mice (Fig. 4A–E). Similarly, the insulin-induced p-Akt (T308) (*P* < 0.05) and p-GSK3β (S9) (*P* < 0.05) levels were also reduced in the GAS of *Ip3r1*^*MKO*^ mice, while the p-IRβ (Y1146) level was markedly increased (*P* < 0.05) (Fig. 4F–J).

### *Ip3r1* deficiency in adipocytes is protected from HFD-induced obesity and metabolic dysfunction

*Ip3r1*^*FKO*^ mice were generated to access the metabolic roles of adipocyte IP3R1. *Ip3r1* mRNA expression levels were significantly decreased in the eWAT and iWAT of *Ip3r1*^*FKO*^ mice (*P* < 0.05), and remained unaltered in other metabolically active tissues (Supplementary Fig. S2A). Further, *Ip3r2* and *Ip3r3* expression levels in iWAT and eWAT of *Ip3r1*^*FKO*^ mice remained indistinguishable from those of WT mice (Supplementary Fig. S2B, C). When fed the regular chow diet, WT and *Ip3r1*^*FKO*^ mice showed similar body weight, body composition, and insulin sensitivity (Supplementary Fig. S2D–H, J), but improved glucose tolerance was observed in *Ip3r1*^*FKO*^ mice (Supplementary Fig. S2I).

**Fig. 5:**
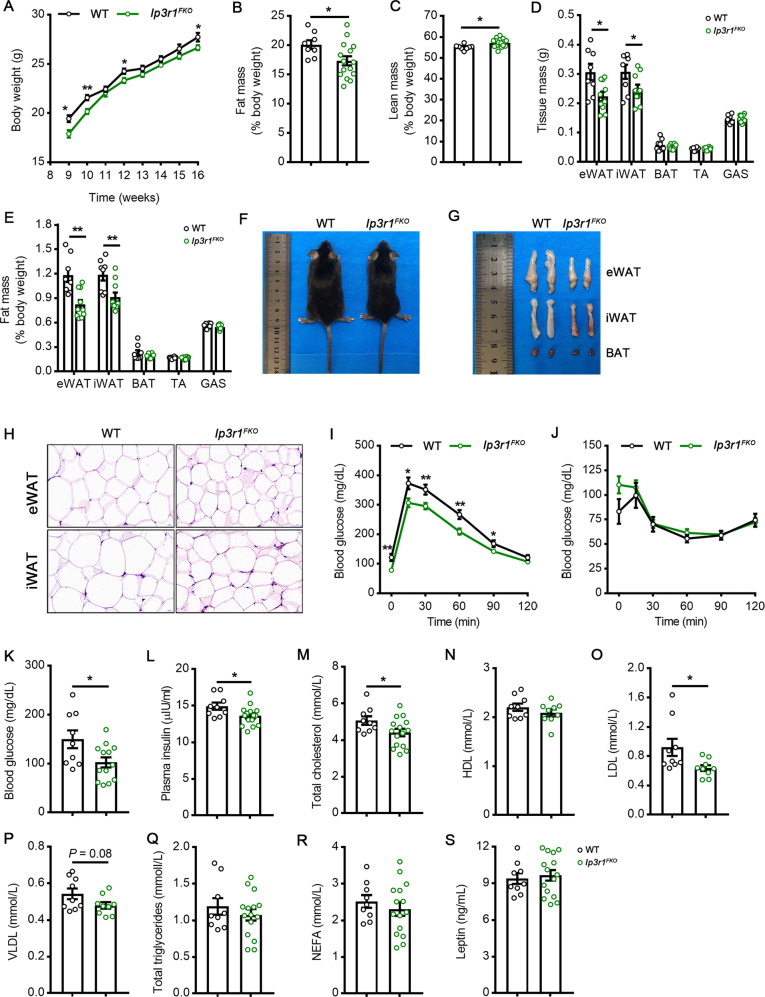
*Ip3r1* specific deletion in adipocytes protects from HFD-induced obesity and metabolic disorders. **A** Body weight measurement of mice maintained on HFD (*n* = 8–15). **B** Fat mass and (**C**) lean mass analyzed by the nuclear magnetic resonance system (*n* = 8–15). **D** Tissue mass (eWAT, iWAT, BAT, TA, and GAS) and (**E**) percentage after 8 wk on HFD (*n* = 8–10). **F** Representative images of WT and *Ip3r1*^*FKO*^ mice (8 wk on HFD). **G** Representative images of adipose tissues (eWAT, iWAT, and BAT) isolated from WT and *Ip3r1*^*FKO*^ mice (8 wk on HFD). **H** Representative images of H&E stained sections of eWAT and iWAT. Scale bar = 20 µm. **I** GTT (2 g/kg glucose, i.p.) (*n* = 8–12). **J** ITT (1U/kg insulin, i.p.) (*n* = 8–12). **K** Blood glucose levels and (**L**–**S**) circulating plasma levels of (**L**) insulin, (**M**) total cholesterol, (**N**) HDL, (**O**) LDL, (**P**) VLDL, (**Q**) total triglycerides, (**R**) NEFA, and (**S**) leptin in WT and *Ip3r1*^*FKO*^ mice (*n* = 8–15). All Data were presented as means ± SEM. ^*^P < 0.05, ^**^P < 0.01. **A**–**H**, **K**–**S**: two-tailed unpaired Student’s *t* test; **I** and **J**: two-way ANOVA followed by Bonferroni’s post hoc test.

**Fig. 6:**
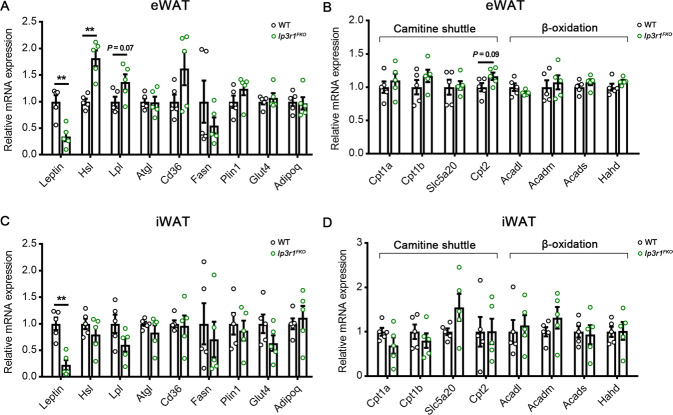
Expression profile of genes related to fatty acid metabolism in white adipose tissues. Relative mRNA expression levels of genes related to lipid synthesis, carnitine shuttle, and β-oxidation in (**A**, **B**) eWAT and (**C**, **D**) iWAT isolated from WT and *Ip3r1*^*FKO*^ mice maintained on a high-fat diet for 8 wk (*n* = 5). All Data were analyzed by two-tailed unpaired Student’s *t* test and presented as means ± SEM. ^**^P < 0.01.

Next, to address the potential role of adipocyte IP3R1 in diet-induced metabolic dysregulation, WT and *Ip3r1*^*FKO*^ mice were maintained on the HFD for 8 wk from 8 wk of age. *Ip3r1*^*FKO*^ mice gained less body weight than WT mice (Fig. 5A), mainly due to a significant reduction in fat mass but not lean mass as revealed by MRI scanning and anatomy data (Fig. 5B–E). Consistently, smaller body size, adipose tissue size (eWAT and iWAT), and adipocyte size were also observed in *Ip3r1*^*FKO*^ mice (Fig. 5F–H). A strikingly improved glucose tolerance and a reduction in blood glucose, plasma insulin, total cholesterol, LDL and VLDL levels were observed in *IP3R1*^*FKO*^ mice compared with those in WT mice (Fig. 5I, K, L, M, O, P). Loss of adipocyte *Ip3r1* had no significant effect on plasma HDL, total triglycerides, NEFA and leptin levels (Fig. 5N, Q–S). Furthermore, mRNA expression levels of *Leptin* were significantly decreased in the eWAT and iWAT of *Ip3r1*^*FKO*^ mice (*P* < 0.01, Fig. 6A, C). *Hsl* expression was also significantly increased in eWAT (*P* < 0.01, Fig. 6A), indicating the enhanced adipokinetic action in eWAT. Expression levels of several genes encoding for the components of carnitine shuttle (*Cpt1a*, *Cpt1b*, *Slc25a20* and *Cpt2*) and involved in β-oxidation (*Acadl*, *Acadm*, *Acads* and *Hadh*) were not affected (Fig. 6B, D). Therefore, a lack of IP3R1 in adipocytes could combat the development of diet-induced obesity, insulin resistance and dyslipidemia. The lack of *Ip3r1* in adipocytes on whole-body energy homeostasis was further examined. The two groups of mice maintained on the HFD showed no difference in physical activity, O_2_ consumption, CO_2_ production, EE and RER (Supplementary Fig. S3B–J). The food intake of *Ip3r1*^*FKO*^ mice was significantly decreased compared with that of WT mice (Supplementary Fig. S3A).

### *Ip3r1*^*FKO*^ mice showed no altered inflammation on HFD

The development of obesity and insulin resistance may be correlated with enhanced peripheral inflammation. Thus, we investigated whether knockout of *Ip3r1* in adipocytes affected peripheral inflammation. F4/80 stained eWAT and iWAT sections from HFD mice were analyzed, and adipose tissues from WT and *Ip3r1*^*FKO*^ mice showed similar infiltration of pro-inflammatory immune cells (Supplementary Fig. S4A). Circulating levels of pro-inflammatory cytokines, including IL4, IL6, IFNγ and MCP1, and anti-inflammatory cytokine IL10 showed no significant differences, and plasma resistin levels tended to increase in *Ip3r1*^*FKO*^ mice (Supplementary Fig. S4B–G). In agreement with plasma cytokine levels, expression levels of *Mcp1*, *Il6*, *Tnfα*, *Ifnγ*, *Mip1α* and *Mip1β* did not significantly change (Supplementary Fig. S4H, I). The mRNA expression levels of macrophage markers (*F4/80* and *Cd68*) were also analyzed. Confusingly, *F4/80*expression was significantly increased in eWAT of *Ip3r1*^*FKO*^ mice (*P* < 0.05, Fig. S4H). These experiments reveal that adipocyte-specific knockout of *Ip3r1* has little impact on obesity-associated inflammation.

### IP3R1 regulates AMPK signaling in white adipose tissues

**Fig. 7:**
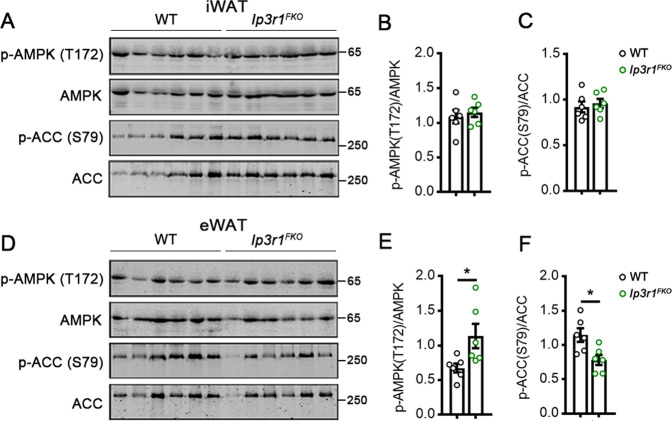
*Ip3r1*^*FKO*^ mice showed activated AMPK signaling in white adipose tissues. Western blot analysis of p-AMPK (T172), p-ACC (S79), AMPK, and ACC in (**A**–**C**) iWAT and (**D**–**F**) eWAT lysates. Quantification of p-AMPK (T172)/AMPK and p-ACC (S79)/ACC was determined by ImageJ software (*n* = 6). Data were analyzed by two-tailed unpaired Student’s *t* test and presented as means ± SEM. ^*^P < 0.05.

To further understand the role of adipocyte IP3R1 in regulating metabolic homeostasis, AMPK signaling was measured by western blotting. Following feeding with HFD for 8 wk, WT and *Ip3r1*^*FKO*^ mice showed similar phosphorylation levels of AMPK and ACC in iWAT (Fig. 7A–C). Strikingly, eWAT from *Ip3r1*^*FKO*^ mice showed increased levels of p-AMPK (T172) and decreased levels of p-ACC (S79) compared to WT mice (Fig. 7D–F). Thus, IP3R1 activates AMPK signaling in visceral fat but not subcutaneous fat in mice offered HFD.

## Discussion

Obesity is one of the main risk factors for developing type 2 diabetes. Maintaining Ca^2+^ homeostasis is critical for the function of metabolic organs [11, 20]. The imbalance of Ca^2+^ also leads to ectopic adipocyte accumulation [21] and obesity-related brown adipose tissue whitening [22]. IP3Rs are key players controlling Ca^2+^ release from ER to cytoplasm or mitochondria. In the current study, we systematically analyzed the role of IP3R1 in glucose homeostasis by making skeletal muscle- or adipocyte-specific knockout mice. Results demonstrated that skeletal muscle *Ip3r1* deletion improved systemic glucose metabolism of mice fed a regular diet but impaired insulin sensitivity in obese mice. Significantly, loss of *Ip3r1* in mature adipocytes enhanced lipolysis and AMPK signaling, especially in the visceral fat, contributing to the improved glucose tolerance in obese mice. An important observation was the increased IP3R1 expression level in the WAT of diet-induced obese mice, revealing the inverse correlation between IP3R1 expression in WAT and glucose homeostasis. Consequently, the result may suggest a dominant contribution by adipose tissue IP3R1 to systemic glucose metabolism in the condition of diet-induced obesity.

Here, we show that *Ip3r1* deletion in skeletal muscle improved glucose tolerance and insulin sensitivity of mice fed regular diets but accelerated insulin resistance in obese mice, which was confirmed by the decreased insulin-mediated Akt signaling in skeletal muscle. Insulin-mediated activation of Akt is central to glucose disposal in mammals [23, 24] and insulin-stimulated Akt phosphorylation is decreased in the skeletal muscle of individuals with insulin resistance [23]. It should be noted that spare insulin receptors are present in metabolic tissues [25]. Normal glucose and insulin tolerance were maintained in mice with heterozygous loss of IR, and Akt signaling was not impaired in skeletal muscle upon IR deficiency [26], suggesting the non-linearity between IR and Akt activity, which could also account for the paradoxical lack of change or increase in IRβ phosphorylation with a corresponding decline in Akt phosphorylation in this study. Notably, *Ip3r1* deletion in skeletal muscle resulted in muscle loss but not weight loss in obese mice, which was in alignment with recent studies that IP3R1 knockdown or inhibition repressed myoblast differentiation [27]. Insulin resistance and muscle loss often coincide in individuals with type 2 diabetes [28], and individuals with low muscle mass have a higher prevalence of metabolic syndrome [29, 30]. Therefore, HFD-induced insulin resistance may be further aggravated by the lower muscle mass in *Ip3r1*^*MKO*^ mice, but the causal relationship between muscle mass and the development of type 2 diabetes remains to be elucidated [31].

Feeding mice an HFD elevated IP3R1 expression in WAT, revealing its potential role in obesity-associated adipocyte pathophysiology. In this study, *Ip3r1* deficiency in adipocytes prevented HFD-induced obesity and adipocyte hypertrophy. In response to over-nutrition, WAT expands by increasing the size of pre-existing adipocytes (hypertrophy) or generating new adipocytes (hyperplasia). In contrast with adipocyte hyperplasia, adipocyte hypertrophy is correlated with pathological WAT remodeling and leads to a deterioration of systemic metabolic health [32], which accounts for the improved glucose tolerance in *Ip3r1*^*FKO*^ mice. Although Ca^2+^ signal pathways are present in human preadipocytes [33], the role of IP3R1 in adipocyte hyperplasia remains unclear yet. Additionally, WAT dysregulation often causes local and systemic inflammation [4]. However, no reduction was observed in plasma cytokine levels in *Ip3r1*^*FKO*^ obese mice, suggesting that IP3R1 activation does not lead to obesity-associated inflammation.

Herein, enhanced lipolysis in WAT, evidenced by the increased *Hsl* expression in eWAT and decreased total triglyceride, LDL and VLDL levels, may contribute to the reduced adiposity. Visceral WAT accumulation is associated with the risk of insulin resistance, whereas subcutaneous WAT expansion is protective due to the differences in location and adipocyte heterogeneity [34]. Therefore, IP3R1 is of great interest for future development as a target to combat the pathological expansion of visceral WAT. In tissues such as WAT, AMPK is a master regulator of energy metabolism. Mice lacking AMPK in adipocytes are susceptible to diet-induced glucose intolerance [35]. Short-term AMPK activation in the liver reduced blood glucose levels and induced fatty acid utilization in adipose tissue [36]. Together, these studies strongly demonstrate that increased AMPK activity in the visceral WAT after *Ip3r1* knockout in adipocytes improves whole-body metabolism. Another interesting observation was the reduced food intake and *Leptin* mRNA expression in eWAT and iWAT of *Ip3r1*^*FKO*^ mice. Although partial leptin deficiency was reported to reduce food intake and protect mice from diet-induced obesity and metabolic disorders [37], it should also be noted that plasma leptin levels remained unchanged in WT and *Ip3r1*^*FKO*^ mice. Future studies still need to address the exact mechanism behind the beneficial effect of *Ip3r1* deficiency in adipocytes. Anyway, this study strongly suggests that IP3R1 antagonists exclusively targeting adipose tissue may be beneficial for the treatment of obesity and type 2 diabetes. Available antagonists, such as Xestospongins and 2-APB, exhibits low specificity or low receptor affinity, and the development of new antagonists of IP3R1 is also challenging due to the ~70% homology of three IP3R subtypes [38]. Considering the structural heterogeneity of IP3Rs in the presence of different activators [39], the structural basis of gating IP3R1 should be thoroughly deciphered first. Additionally, combined pharmacophore and grid-independent molecular descriptors (GRIND) analysis can be applied to screen potential antagonists against IP3R1 as described previously [40].

Some limitations of this study should also be noted. As *Ip3r1* deficiency was achieved in skeletal muscle and adipocytes, we can’t confirm it at the protein level due to its low expression. The understanding of IP3R1 in adipocyte precursors (APs) is still limited. AP-specific *Ip3r1* knockout mice could be generated by mating *Ip3r1*-floxed mice with *Pdgfrα*-Cre mice to address this issue. Besides, although *Ip3r1* deficiency in adipocytes enhanced AMPK signaling in the visceral fat, β-oxidation of fatty acids was not influenced. A comprehensive analysis is needed to identify the metabolic landscape regulated by IP3R1. In addition, *Ip3r1* knockout in adipocytes reduced the food intake of obese mice, and we cannot rule out the possibility that the reduced food intake led to lipolysis. Collectively, our study reveals that IP3R1 in skeletal muscle and adipocytes exerts divergent effects on obesity and obesity-related metabolic disorders and provides a rational basis for developing adipocyte IP3R1 as a promising target for the treatment of obesity and type 2 diabetes.

## Materials and methods

### Animal model

All mice used in this study were C57BL/6 background. To obtain mice with a conditional knockout allele of *Ip3r1*, exon 3 was selected as conditional knockout (cKO) region and flanked by *loxp* sites (referred to as floxed) using gene targeting in C57BL/6 embryonic stem (ES) cells and built by Cyagen Biosciences (Guangzhou, China). The procedure of knockout mice generation in this study was similar to that described previously [41], including construction of targeting vector, electroporation of ES cells, G418 selection, identification of homologous recombined ES cells, and generation of *Ip3r1*^*f/f*^ mice. To generate mice lacking IP3R1 selectively in skeletal muscle, IP3R1-floxed mice were crossed with *Myf5-cre* mice (007893, Jackson Laboratory) expressing recombinase under the control of Myf5 promoter. Mice used for experiments were *Ip3r1*^*f/f*^ (wild type, WT) and *Myf5-cre*^*+/-*^*Ip3r1*^*f/f*^ (*Ip3r1*^*MKO*^) mice. To generate mice lacking IP3R1 selectively in adipocytes, we crossed IP3R1-floxed mice with *Adipoq-cre* mice expressing recombinase under the control of Adiponectin promoter. *Adipoq-cre* mice were purchased from the Jackson Laboratory (J010803). Mice used for experiments were *Ip3r1*^*f/f*^ (WT) and *Adipoq-cre*^*+/−*^*Ip3r1*^*f/f*^ (*Ip3r1*^*FKO*^) mice.

### Mouse maintenance and diet-induced obesity

Mice were kept in a temperature-controlled environment (23 ± 2 °C) and had free access to food and water under a 12 h/12 h light/dark cycle. Except for obesity, the mice were in generally good health. To induce obesity, 4 or 8-week-old male mice were fed a high-fat diet (60% kcal fat, H10060, Huafukang Bioscience, Beijing, China) for 8 week, and mice received a chow diet (10% kcal fat, H10010, Huafukang Bioscience, Beijing, China) were served as control. Adult male mice were used in this study unless specified. Genotype- and sex-matched mice were randomly assigned to experimental groups mitigating the cage effect and no blinding was done in this study.

### Glucose and insulin tolerance tests

Glucose tolerance test (GTT) and insulin tolerance test (ITT) were carried out in the morning after a 6 h of fasting. After determining the fasted blood glucose levels, mice were intraperitoneally injected with glucose (2 g/kg body weight) or insulin (1 U/kg body weight) for GTT and ITT, respectively. At 15, 30, 60, 90 and 120 min after injection, blood was collected from the tail tip and glucose concentrations were determined using contour portable glucometer (Sinocare, Changsha, China). All GTT and ITT were performed on adult mice that were more than 8-week-old. To ensure that stress was minimized prior to and during these tests, experimental mice were handled at least once every week after weaning [42].

### Plasma metabolic profiling and cytokine levels

Mice at the fed state or fasted for 12 h were sacrificed, and whole blood was collected from retrobulbar venous plexus and centrifuged for 10 min at 12,000 × *g* to obtain plasma. Plasma glucose, total cholesterol, high-density lipoprotein (HDL), low-density lipoprotein (LDL), very low-density lipoprotein (VLDL), and total triglycerides were measured using commercial kits (Nanjing Jiancheng Bioengineering Institute, Nanjing, China). ELISA kits (Sinoukbio, Beijing, China) were used to measure plasma insulin, C-peptide, non-esterified fatty acid (NEFA), leptin, IL (interleukin) 4, IL6, IL10, resistin, interferon γ (IFNγ) and monocyte chemotactic protein-1 (MCP1) following the manufacturer’s instruction.

### Body composition analysis

Body composition, including whole-body fat mass and lean mass, were analyzed by a nuclear magnetic resonance system (Body Composition Analyzer QMR06-090H, Niumag Corporation, Shanghai, China).

### Indirect calorimetry

Energy expenditure (O_2_ consumption/CO_2_ production), locomotor activity, and food intake were determined by metabolic cages using an Oxylet system (Columbus Instruments, Columbus, USA). Mice were individually housed in metabolic chambers with food and tap water ad libitum. The sampling interval for each cage was 3 min, with repetition every 27 min. Oxygen consumption (VO_2_), carbon dioxide production (VCO_2_), and spontaneous motor activity were measured over three consecutive days. Expiratory exchange ratio (RER) was calculated by VCO_2_/VO_2_.

### Histology of skeletal muscle and adipose tissues

All tissues analyzed in this study were collected in the fasted state. Different skeletal muscle and adipose tissues were dissected from mice and quickly fixed in 4% paraformaldehyde. Samples embedded in paraffin were sectioned into transverse sections with a thickness of 5 μm, followed by haematoxylin and eosin (H&E) staining. Muscle fiber cross-sectional area was determined by Adobe Photoshop (CS6 version, Adobe Systems Inc., San Jose, USA). Fixed adipose tissues were stained for F4/80 using standard immunohistochemistry methods as described previously [43].

### RNA extraction and qPCR

Total RNA was isolated from frozen tissues or organs using RNAiso Plus (Takara Biomedical Technology, 9108, Beijing, China) and reverse-transcribed into cDNA using a PrimeScript RT reagent kit with gDNA Eraser (Takara Biomedical Technology, RR047A, Beijing, China). SYBR Green-based qPCR was performed in a qTOWER 2.2 thermocycler (Analytik Jena, Jena, Germany). The mRNA expression levels of target genes were normalized to that of *Gapdh*. The primer sequences for qPCR were listed in Supplementary Table S1.

### Western blot assay

Skeletal muscle or adipose tissues were lysed in RIPA lysis buffer (Huaxingbio, Beijing, China) with a protease inhibitor cocktail (Roche, Basel, Switzerland). Approximately 60 μg of total protein was resolved on 8–10% SDS-PAGE gels and transferred to polyvinylidene fluoride membranes (Millipore, Boston, USA). Membranes were blocked in TBS containing 5% (w/v) bovine serum albumin at room temperature for 1 h and then incubated against primary antibodies (Supplementary Table S2) at 4 °C overnight. Blots were developed using DyLight 800-labeled secondary antibodies, detected with the Odyssey Clx (4647 Superior Street, LI-COR Biotechnology, Lincoln, NE) and quantified by ImageJ software (National Institutes of Health, Bethesda, USA).

### Statistics

All data were analyzed in SPSS software (IBM SPSS Statistics 23) and presented as means ± SEM. The number of mouse samples per group was 3–15. The exact sample size for each experimental group/condition was given as a number in the figure legends. Normal distribution of populations at 0.05 level was calculated using Shapiro–Wilk Test. Data were tested by two-way ANOVA followed by Bonferroni’s post hoc test or two-tailed unpaired Student’s *t* test. The test applied and n were stated in the Figure Legend. A value of *P* < 0.05 was considered significant (^*^P < 0.05, ^**^P < 0.01) and 0.05 ≤ *P* ≤ 0.10 was considered to have a trend.

## Supplementary information

Supplementatary Information

Original western blots

## Data Availability

The datasets used and analyzed during the current study are available from the corresponding author on reasonable request.
