# Supplementary material for: Lack of adipocyte IP3R1 reduces diet-induced obesity and greatly improves whole-body glucose homeostasis
Source: Cell Death Discov. 2023 Mar 9;9:87. doi: 10.1038/s41420-023-01389-y (PMC9998023; doi:10.1038/s41420-023-01389-y)
Supplement: Supplementary file 1 — Supplementatary Information [file 41420_2023_1389_MOESM1_ESM.docx]

**Lack of adipocyte IP3R1 reduces diet-induced obesity and greatly improves whole-body glucose homeostasis**

Xin Zhang^1,2,#^, Lu Wang^1,#^, Yubo Wang^1^, Linjuan He^1^, Doudou Xu^1^, Enfa Yan^1^, Jianxin Guo^1^, Chenghong Ma^1^, Pengguang Zhang^1^, Jingdong Yin^1,2*^

^1^ State Key Laboratory of Animal Nutrition, College of Animal Science and Technology, China Agricultural University, Beijing, 100193, China

^2^ Molecular Design Breeding Frontier Science Center of the Ministry of Education, Beijing, 100193, China

^#^ These authors contributed equally to this work.

^*^ Correspondence: Jingdong Yin; State Key Laboratory of Animal Nutrition, College of Animal Science and Technology, China Agricultural University, Beijing, China; 8610-62733590-1404; E-mail: [yinjd@cau.edu.cn](mailto:yinjd@cau.edu.cn).

**
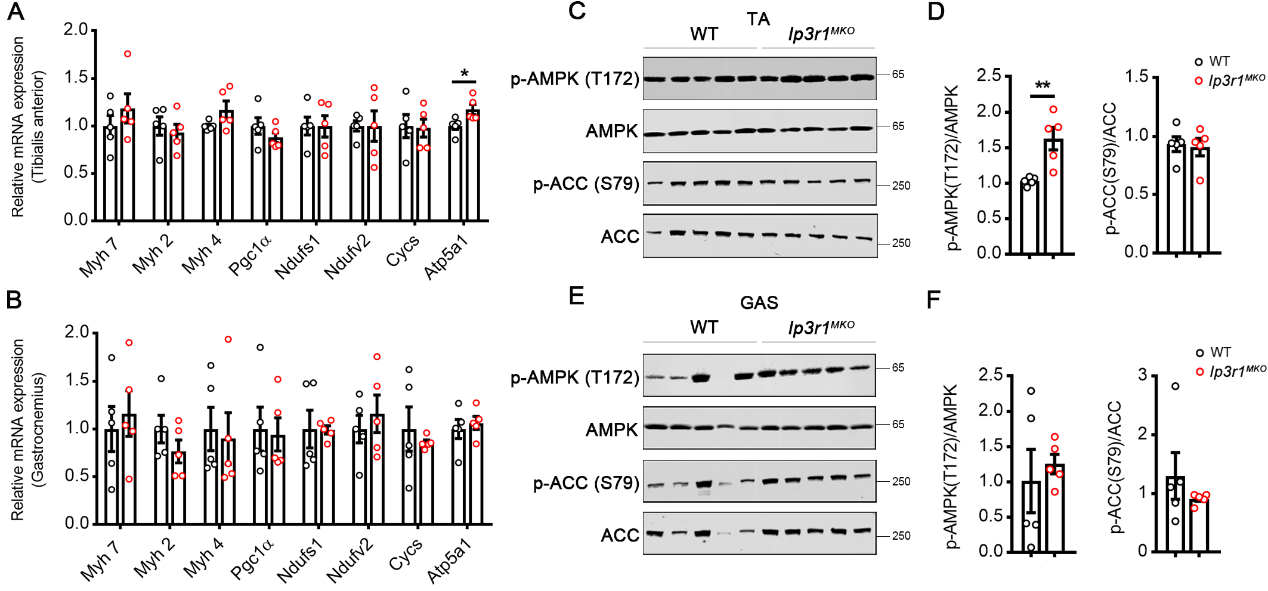
**

**Figure S1. Metabolic analysis of WT and *Ip3r1^MKO^* mice maintained on high fat diet.**

(A-B) Relative mRNA expression levels of muscle fiber-type and mitochondrial/oxidative phosphorylation markers in TA and GAS (n = 5). Western blot analysis of p-AMPK (T172), AMPK, p-ACC (S79) and ACC in (C-D) TA and (E-F) GAS lysates isolated from WT and *Ip3r1^MKO^* mice. Quatification of p-AMPK (T172)/AMPK and p-ACC (S79)/ACC was determined by ImageJ software (n = 5). All data were analyzed by two-tailed unpaired Student’s *t* test and presented as means ± SEM. ^*^*P* < 0.05, ^**^*P* < 0.01.


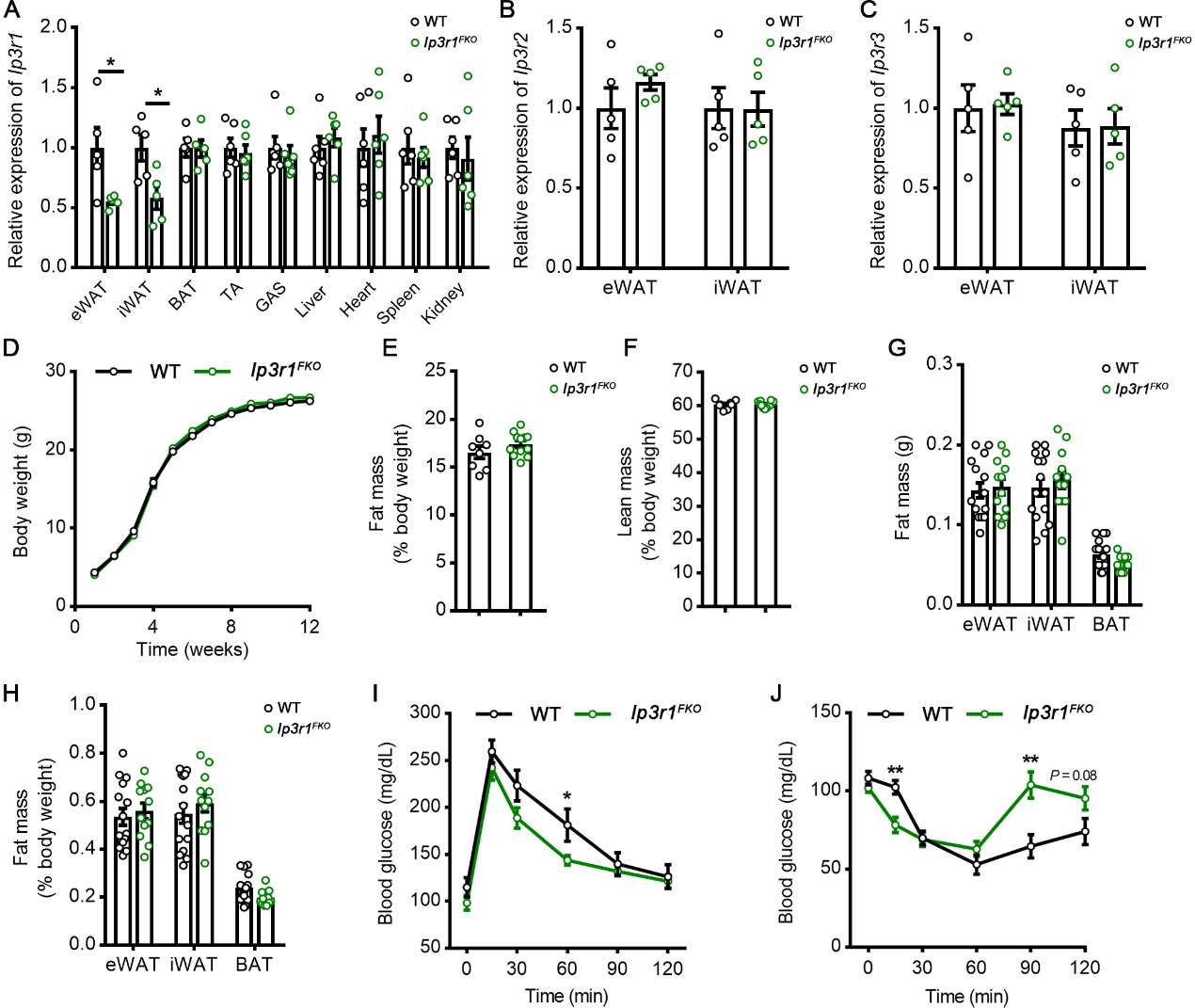


**Figure S2. Body composition and glucose homeostasis of WT and *Ip3r1^FKO^* mice maintained on chow diet.**

(A) mRNA levels of *Ip3r1* in adipose tissues (eWAT, iWAT, and BAT), skeletal muscles (TA and GAS), liver, heart, spleen and kidney of WT and *IP3R1^FKO^* mice (n = 5). (B-C) mRNA levels of *Ip3r2* and *Ip3r3* in eWAT and iWAT of WT and *IP3R1^FKO^* mice (n = 5). (D) Body weight measurement on chow diet (n = 12-15). (E) Fat mass and (F) lean mass analyzed by the nuclear magnetic resonance system (n = 8-10). (G) Weight and (H) weight percentage of the body of eWAT, iWAT and BAT (n = 12-15). (I) GTT (2g/kg glucose, i.p.) (n = 12-15). (J) ITT (1U/kg insulin, i.p.) (n = 12-15). Data were presented as means ± SEM. ^*^*P* < 0.05. ^**^*P* < 0.01. A-H: two-tailed unpaired Student’s *t* test; I and J: two-way ANOVA followed by Bonferroni’s post hoc test.


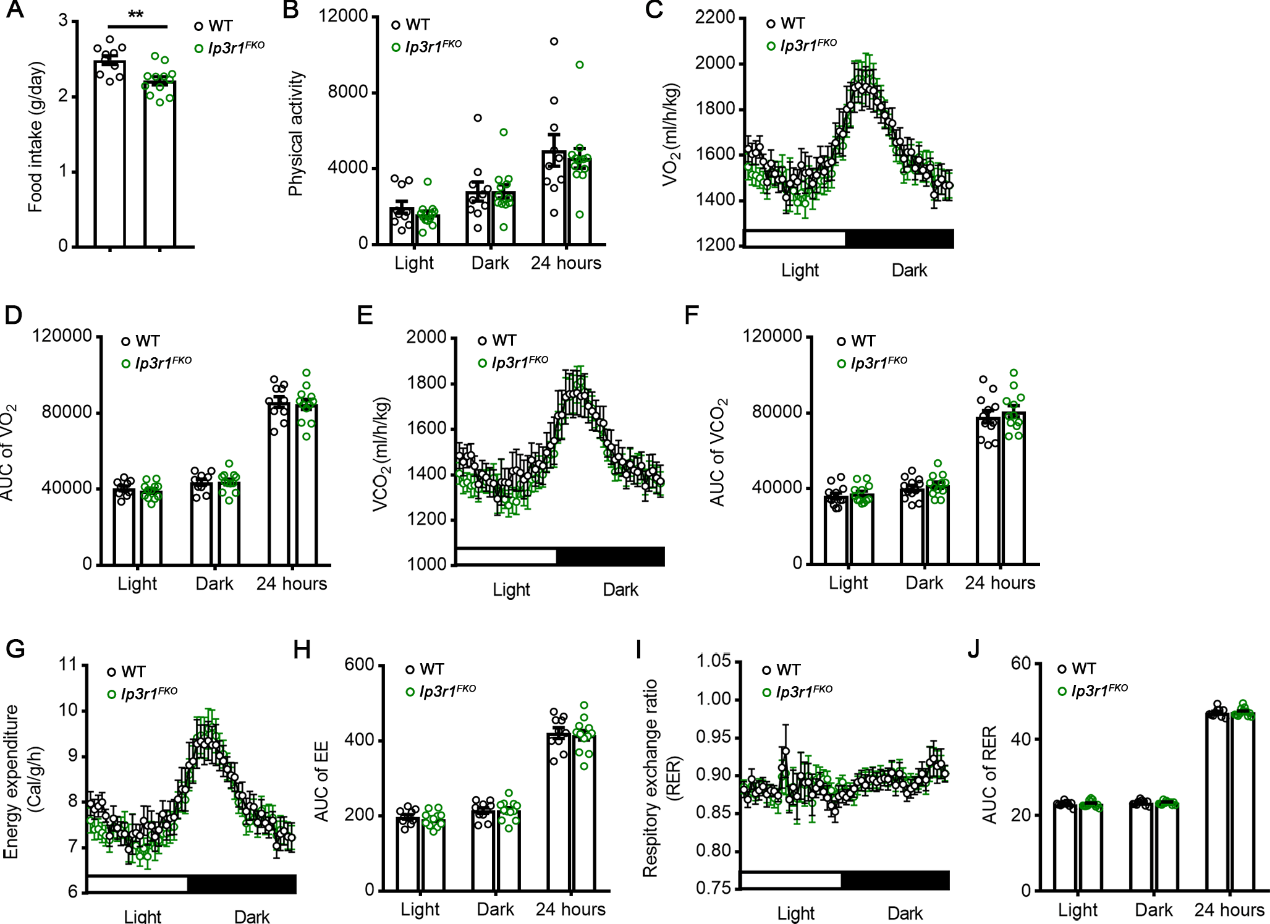


**Figure S3. Metabolic analysis of WT and *Ip3r1^FKO^* mice maintained on high fat diet.**

(A) Food intake. (B) Physical activity. (C-D) Oxygen consumption rate (VO_2_). (E-F) Carbon dioxide production rate (VCO_2_). (G-H) Energy expenditure (EE). (I-J) Respiratory exchange ratio (RER). n = 9-12. All Data were analyzed by two-tailed unpaired Student’s *t* test and presented as means ± SEM. ^**^*P* < 0.01.


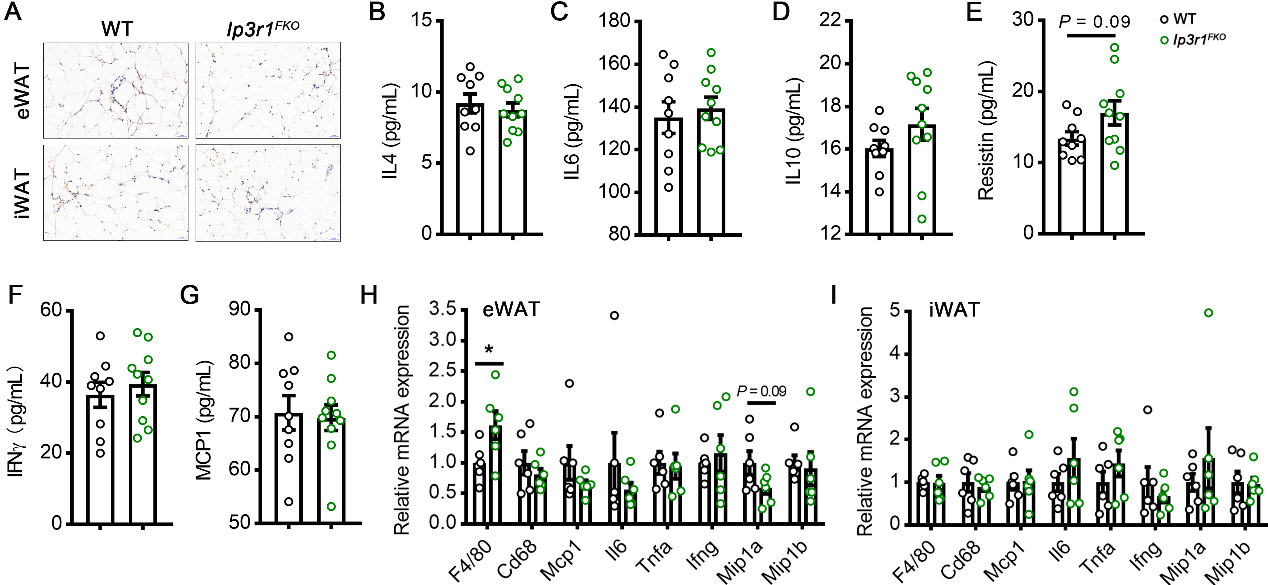


**Figure S4. *Ip3r1^FKO^* mice show no difference on HFD-induced peripheral inflammation.**

(A) Representative F4/80-stained sections of eWAT and iWAT from WT and *Ip3r1^FKO^* mice. Scale bar = 50 μm. Circulating plasma levels of (B) IL4, (C) IL6, (D) IL10, (E) resistin, (F) IFNγ, and (G) MCP1 in WT and *Ip3r1^FKO^* mice (n = 9-10). Relative mRNA expression levels of inflammation markers in (H) eWAT and (I) iWAT of WT and *Ip3r1^FKO^* mice maintained on HFD (n = 6). All Data were analyzed by two-tailed unpaired Student’s *t* test and presented as means ± SEM. ^*^*P* < 0.05.

**Table S1. List of primers used for qPCR assay**

| Gene name | Gene ID | Forward (5’-3’) | Reverse (5’-3’) | Annealing temperature (℃) | Product size (bp) |
| --- | --- | --- | --- | --- | --- |
| *Ip3r1* | 16438 | TGGCAGAGATGATCAGGGAAA | GCTCGTTCTGTTCCCCTTCAG | 60 | 96 |
| *Ip3r2* | 16439 | GAGGCAAATGAGGAATCCAA | TGCTGTCGATCTGAGGTGTC | 60 | 70 |
| *Ip3r3* | 16440 | CCCAGCTACAAGGGTGATGT | GCCACCAGACGGTACTTGAT | 60 | 107 |
| *Myh7* | 140781 | GTCAAGGCCAAGATCGTGTC | CTCCTTCACAGTCACCGTCT | 60 | 81 |
| *Myh2* | 17882 | CAGTGTCTAAGGCCAAGGGA | TCTCATCAAGCTGCCTGGAA | 60 | 171 |
| *Myh4* | 17884 | AAGCCTGCCTCCTTCTTCAT | CAAACACCGATGACTTGGCA | 60 | 192 |
| *Pgc1α* | 19017 | AGCCGTGACCACTGACAACGAG | GCTGCATGGTTCTGAGTGCTAAG | 58 | 168 |
| *Ndufs1* | 227197 | AGGATATGTTCGCACAACTGG | TCATGGTAACAGAATCGAGGGA | 60 | 147 |
| *Ndufv2* | 72900 | GCAAGGAATTTGCATAAGACAGC | TAGCCATCCATTCTGCCTTTG | 60 | 216 |
| *Cycs* | 13063 | AAATCTCCACGGTCTGTTCGG | GGGTATCCTCTCCCCAGGTG | 60 | 101 |
| *Atp5a1* | 11946 | CATTGGTGATGGTATTGCGC | TCCCAAACACGACAACTCC | 60 | 134 |
| *Leptin* | 16846 | CAAGCAGTGCCTATCCAGA | AAGCCCAGGAATGAAGTCCA | 60 | 141 |
| *Hsl* | 16890 | AGTTCCCTCTTTACCGGTGG | ACGACAGCACCTCAATCTCA | 60 | 136 |
| *Lpl* | 16956 | TTCAACCACAGCAGCAAGAC | CTGGATAATGTTGCTGGGCC | 60 | 163 |
| *Atgl* | 66853 | CAACGCCACTCACATCTACG | ACCAGGTTGAAGGAGGGATG | 60 | 159 |
| *Cd36* | 12491 | TCATATTGTGCTTGCAAATCCAA | TGTAGATCGGCTTTACCAAAGATG | 60 | 91 |
| *Fasn* | 14104 | GCTTCGCCAACTCTACCATG | CCATCGCTTCCAGGACAATG | 60 | 117 |
| *Plin1* | 103968 | GAGAGGAGACAGACGACGAG | GGCTGTAACCTCTCTGAGCA | 60 | 74 |
| *Adipoq* | 11450 | TACTGCAACATTCCGGGACT | GTAGGTGAAGAGAACGGCCT | 60 | 108 |
| *Cpt1a* | 12894 | GACGAATCGGAACAGGGATA | TGGCATAGCTGTCAATAGATGC | 58 | 61 |
| *Cpt1b* | 12895 | GAGTGACTGGTGGGAAGAATATG | GCTGCTTGCACATTTGTGTT | 58 | 114 |
| *Slc25a20* | 57279 | TGAAGGCCCTGTTACACTCA | CCTCCAGAGAGTCAGCCATC | 58 | 62 |
| *Cpt2* | 12896 | CCAAAGAAGCAGCGATGG | TAGAGCTCAGGCAGGGTGA | 58 | 94 |
| *Acadl* | 11363 | GCTTATGAATGTGTGCAACTCC | CCGAGCATCCACGTAAGC | 60 | 81 |
| *Acadm* | 11364 | AGTACCCTGTGGAGAAGCTGAT | TCAATGTGCTCACGAGCTATG | 60 | 100 |
| *Acads* | 11409 | TCTTCCCCACAGCTCAGGT | GTAATCCAAGCCTGCACCA | 58 | 92 |
| *Hadh* | 15107 | CTTGCGCTCCATGTCCTC | ACTACTGTATGGCCAGTTGCTG | 60 | 138 |
| *F4/80* | 13733 | TCCTGCTGTGTCGTGCTGTTC | GCCGTCTGGTTGTCAGTCTTGTC | 62 | 121 |
| *Cd68* | 12514 | TGTCTGATCTTGCTAGGACCG | GAGAGTAACGGCCTTTTTGTGA | 62 | 75 |
| *Mcp1* | 20296 | CCCAATGAGTAGGCTGGAGA | TCTGGACCCATTCCTTCTTG | 58 | 125 |
| *Il6* | 16193 | GGAGCCCACCAAGAACGATA | ACCAGCATCAGTCCCAAGAA | 62 | 98 |
| *Tnfα* | 21926 | TTGTTGCCTCCTCTTTTGCT | TGGTCACCAAATCAGCGTTA | 58 | 81 |
| *Ifnγ* | 15978 | CGGCCTAGCTCTGAGACAAT | AACAGCCAGAAACAGCCATG | 62 | 79 |
| *Mip1a* | 20302 | TGAGAGTCTTGGAGGCAGCGA | TGTGGGTACTTGGCAGCAAACA | 58 | 135 |
| *Mip1b* | 20303 | CTCCCACTTCCTGCTGTTTC | GTCTGCCTCTTTTGGTCAGG | 58 | 126 |
| *Gapdh* | 14433 | GTGTTCCTACCCCCAATGTG | CTTGCTCAGTGTCCTTGCTG | 60 | 349 |

**Table S2. List of antibodies used for western blot assay**

| Antibodies | Source | Identifier |
| --- | --- | --- |
| Mouse monoclonal anti-IRβ | Cell Signaling Technology | Cat#3020 |
| Rabbit polyclonal anti-p-IRβ (Y1146) | Cell Signaling Technology | Cat#3021 |
| Rabbit polyclonal anti-Akt | Cell Signaling Technology | Cat#9272 |
| Rabbit monoclonal anti-p-Akt (S473) | Cell Signaling Technology | Cat#4060 |
| Rabbit monoclonal anti-p-Akt (T308) | Cell Signaling Technology | Cat#2965 |
| Rabbit monoclonal anti-GSK3β | Cell Signaling Technology | Cat#5676 |
| Rabbit monoclonal anti-p-GSK3β (S9) | Cell Signaling Technology | Cat#8566 |
| Rabbit polyclonal anti-AMPKα | Cell Signaling Technology | Cat#2532 |
| Rabbit monoclonal anti-p-AMPKα (T172) | Cell Signaling Technology | Cat#2535 |
| Rabbit polyclonal anti-ACC | Cell Signaling Technology | Cat#3662 |
| Rabbit polyclonal anti-p-ACC (S79) | Cell Signaling Technology | Cat#3661 |
| Rabbit polyclonal anti-IP3R1 | Alomone Labs | Cat#ACC-019 |
| Rabbit polyclonal anti-p-IP3R1 (Y353) | Affinity Biosciences | Cat#AF8342 |
| Rabbit monoclonal anti-β-actin | Cell Signaling Technology | Cat#4970 |
| Rabbit monoclonal anti-GAPDH | Cell Signaling Technology | Cat#2118 |
| Rabbit monoclonal anti-β-tubulin | Cell Signaling Technology | Cat#2128 |
