# Supplementary material for: Lack of adipocyte IP3R1 reduces diet-induced obesity and greatly improves whole-body glucose homeostasis
Source: Cell Death Discov. 2023 Mar 9;9:87. doi: 10.1038/s41420-023-01389-y (PMC9998023; doi:10.1038/s41420-023-01389-y)
Supplement: Supplementary file 2 — Original western blots [file 41420_2023_1389_MOESM2_ESM.pdf]

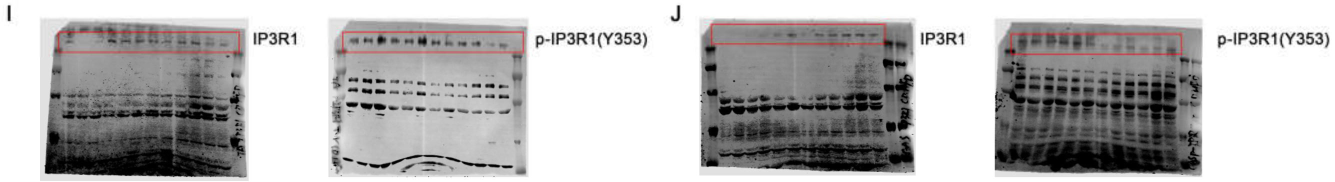

Figure 1. Metabolic analysis and IP3R1 expression of C57BL/6 mice on chow diet (CD) or high-fat diet (HFD).

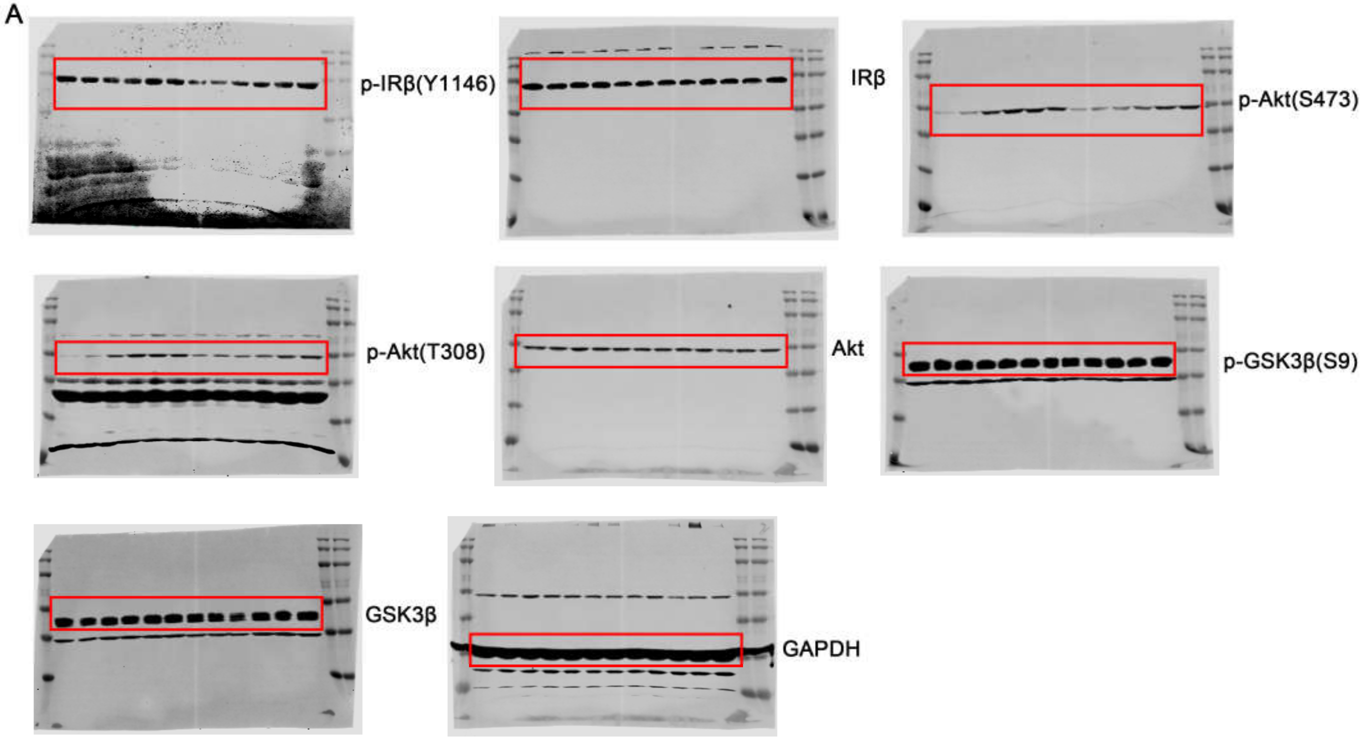

Figure 2. *Ip3r1* deletion in skeletal muscle leads to lower muscle weight.

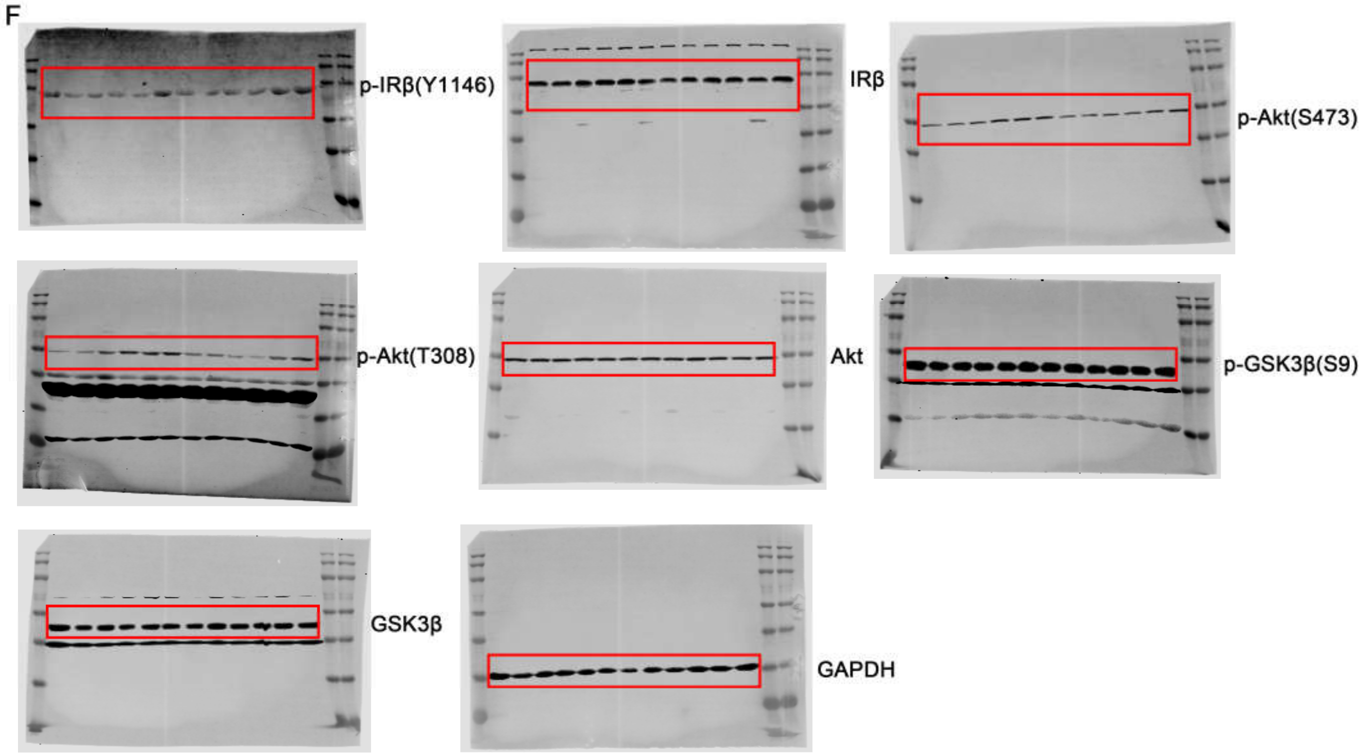

Figure 2. *Ip3r1* deletion in skeletal muscle leads to lower muscle weight.

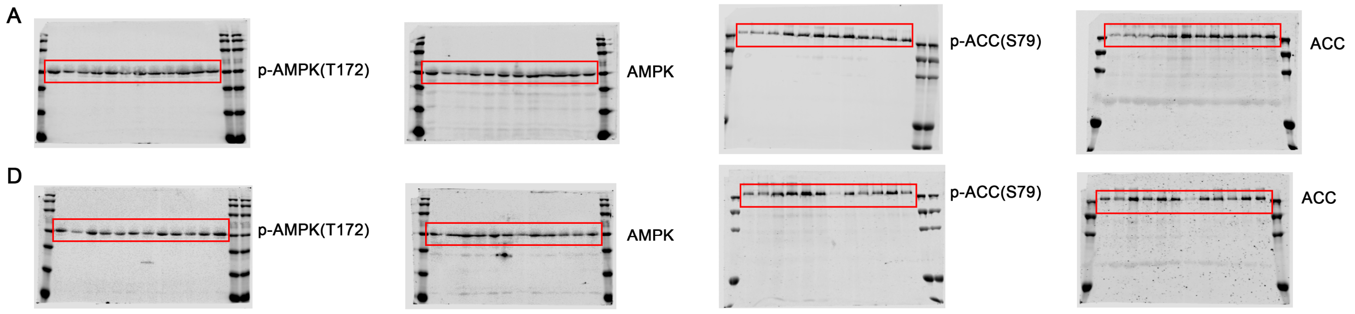

Figure 7. *Ip3r1*^{FKO} mice showed activated AMPK signaling in white adipose tissues.

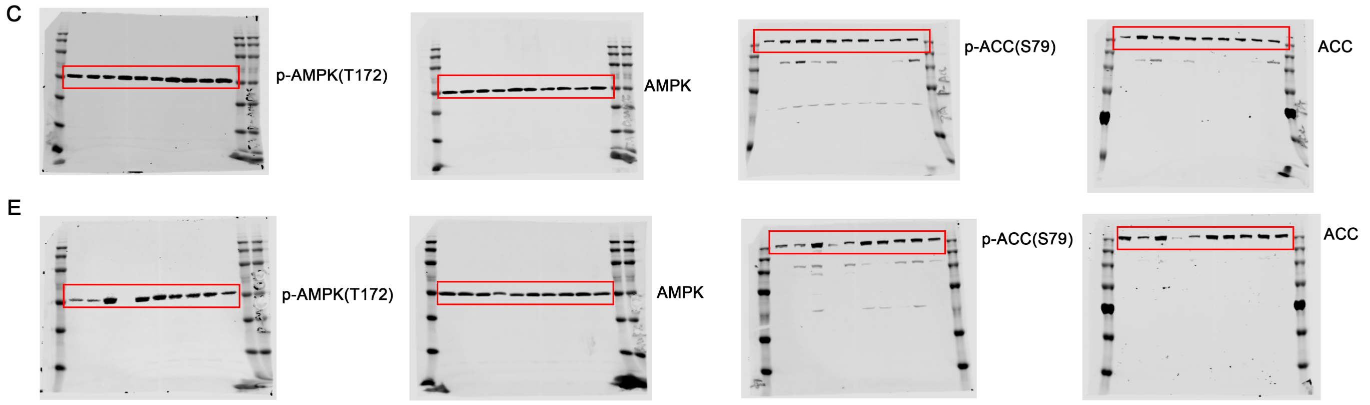

Figure S1. Metabolic analysis of WT and *Ip3r1*^{MKO} mice maintained on high fat diet.
